# Medical Inspection of Schools

**Published:** 1902-07

**Authors:** De Lancey Rochester

**Affiliations:** Buffalo, N. Y., Associate Professor of Principles and Practice of Medicine, University of Buffalo.


### BUFFALO MEDICAL JOURNAL.

VOL. XLI.—LVII. JULY, 1902. NO. 12.

### ORIGINAL COMMUNICATIONS.

### Medical Inspection of Schools.

BY DE LANCEY ROCHESTER, A. B., M. D., Buffalo, N. Y.,
Associate Professor of Principles and Practice of Medicine, University of Buffalo.

THERE are two great duties incumbent upon the members of the medical profession, viz.: the prevention of the occurrence of disease and the treatment of disease when it does occur. In the mind of the layman and, alas, of many of the profession, the second, the treatment of disease—is the chief concern of the physician and in the first he has too little interest.

Nevertheless, ever since scientific medicine, the daughter of the microscope, has had an existence, the chief aim of the most devoted of the worshipers at her shrine has been to enlarge more and more the scope of the first of these duties, so that now preventive medicine is gaining more and more adherents and we may look forward with assured faith that the time will come when the prevention of disease will be the chief—even though it may possibly never be the only—duty of the members of the medical profession.

There is one class of diseases—in which are included some of the most fatal—which, in the opinion of the writer, will at no distant future be put at the bottom instead of at the top of the list of most prevalent diseases: and that class is the class of infectious diseases. While in a large number of these diseases the specific cause is still unknown, in some of them it is known; and in all, whether the specific cause is known or not, the other etiological factors are known and among these factors the transmissibility of the disease from one individual to another is well recognised.

Many of these infectious diseases occur most frequently among children and unfortunately they are often exceedingly serious—even when not fatal, leaving sequelæ that tend to handicap the individual in the race of life.

The question arises as to why these diseases are more common among children; and there are three reasons usually given in answer to that question. The first (and it is doubtful whether it be true) is that the resisting power of the growing organism is less than that of the adult; the second is that as most infectious diseases confer immunity from that disease, adults do not take the diseases because they have had them in childhood. There is a certain amount of truth in this reason, but the fact of the recurrence of these diseases when the exposure is sufficient, detracts greatly from its value. The third and most important reason is that great numbers of children are almost constantly exposed to the dangers of infection through their gathering together day after day in the same rooms in schools. If the first of the reasons has truth in it, that the growing organism has less resisting power to the onslaught of infection than the adult, think what a crime it is to expose these non-resisting individuals to almost daily contact with infection when by a little foresight it might be prevented.

There is a period at the beginning of most infectious diseases when there is the least danger of transmission to others, when, however, the physician can readily recognise the disease. This fact is the main basis of the plea for the value of the establishment of a proper daily medical inspection of schools, public, parochial and private. The ideal hygienic inspection of schools would include the investigation of the sanitary state of the school building and its surroundings; the surroundings as bearing on the purity of the outside air in the neighborhood and as bearing on the purity of the morals of the pupils, as for example the proximity of saloons, low theatres or other vicious resorts; the heating, ventilation and sewering of the schoolhouse; the relation of the light to the pupils in the study and recitation rooms; the condition of the coat rooms where the children hang their outer garments—valuable suggestion has been made that separate wardrobes be provided for the individual clothing—just enough partition to keep the clothes from contact with each other, as a means of preventing spread of infection—and finally the daily inspection of the children to determine whether they are well enough to attend school and whether, if sick, there is danger of transmitting the disease to others. These briefly are the objects to be attained by careful hygienic inspection of schools. Some of them require the attention of those trained in sanitary science, chemistry and architecture. That which distinctly belongs to the physician is the inspection of the children for the detection of defect or disease.

According to Dr. William D. Byrne, of Chicago, in an article published in the *Journal of the American Medical Association*, November 23, 1901, pp. 1 and 2:

This great aid to prophylaxis was foreshadowed in those admirable German laws which went into effect July 14, 1884, by the adoption of the Prussian Circular to the Royal Governors of Provinces, which concerns the closing of schools in the event of the occurrence of infectious diseases. The diseases and the laws pertaining to them are classified as follows:

1.–*a*. Cholera, dysentery, measles, rubella, scarlet fever, diphtheria, smallpox, typhus, relapsing fever, cerebrospinal meningitis; *b*. typhoid fever, contagious inflammation of the eyes, itch and pertussis (while spasmodic).

2. Children suffering from these diseases are to be excluded from school.

3. Healthy children living in same house in which cases named in 1 exist. Exception: it must then be certified by a physician that the school child is protected from danger of infection by proper separation.

4. Children excluded from school for said causes may be readmitted only after the danger of infection has been removed according to a physician's certificate of the usual duration of the disease has expired. This, in the case of scarlet fever and smallpox, is six weeks, and for measles and rubella four weeks, from its inception. Regard must be paid to the thorough cleansing of the child's person and clothing before readmission to school.

5. For the observance of orders 2 and 4 the principal or headmaster is made responsible. An immediate report of the exclusion of a child from school on account of an infectious disease must always be made to the local police authorities.

6. Refers to isolation of children attending boarding school when an epidemic occurs. It requires a physician's certificate to release them from quarantine, which he will give only when convinced that the danger of spreading disease is past and all precautions have been observed.

7. When any person living in the schoolhouse falls sick of one of the infectious diseases named in 1, or if a case should occur in the household of a school teacher, said case must be reported to the principal and to the police authorities, when proper steps must be taken to isolate the infectious case.

8. When several cases of infectious diseases, as in 1, appear in the locality where the school is situated, the principal and teachers must direct their especial attention to the purification of the school property, to the thorough airing of the class rooms. In particular must the school rooms and sanitaries be carefully cleansed each day. School rooms are to be aired continuously when not in use and the sanitaries must be regularly disinfected according to the regulations of the district police authorities. This rule applies to boarding-schools and in them is extended to include dwelling rooms, work rooms and bedrooms of the pupils.

9. School rooms and entire schools where infectious diseases appear in epidemic form must be closed when the district physician advises.

10. Reopening is permissible only after thorough previous cleansing and disinfecting of the premises and on the advice of the district physician.

11. These laws apply as well to parochial and private schools, orphanages, asylums, day missions, and kindergartens.

More important than the closing of schools where an epidemic is raging is the prevention of the epidemic through regular daily medical inspection of pupils. According to Dr. Byrne, "the first attempt at this work was made in the parochial schools of Philadelphia in 1889, but was discontinued on account of parental objection. To Dr. S. H. Durgin, of Boston, is due the credit of the first victory in this field. Beginning to agitate the question in 1890, he encountered much opposition but, aided by Dr. C. M. Green, a member of the School Board, he succeeded in overcoming it . . . . . . His important victory dates from November 1, 1894. In March, 1898, the medical inspection of school was regularly instituted in New York, and in January, 1900, in Chicago and Philadelphia." The following is an extract from the report of the Superintendent of Public Schools of the City of Boston, published in March, 1900, pp. 38-43.

For five and a half years past our schools have received the benefit of regular daily medical inspection. Competent physicians appointed by the Board of Health visit all the schools soon after the opening of the morning session each day. Every class teacher reports to the principal, early in the session, on the condition of the children in the class. If any children seem to be ailing in any way the inspector's attention is called to them. He examines them. If a child is found to be ill, but without manifesting any symptoms of an infectious disease, the teacher is *advised* to send the child home, with a message, written or oral, as may seem best, stating what the trouble may be and suggesting, if medical care seems to be needed, that the family physician be called. The inspector does not declare his diagnosis of the case nor give professional advice as to its treatment. To do so would be to encroach on the province of the family physician, a thing which the inspectors are particularly required to refrain from doing. There are, of course, certain minor ills, pediculosis, for example, not requiring professional advice, for the treatment of which suggestions may be conveyed to the parents directly. Nor would the medical inspector refuse immediate treatment in cases of emergency.

In respect to all non-infectious diseases the medical inspector acts by permission of the school authorities as a friendly adviser. But if he finds a child manifesting symptoms of an infectious disease he at once acts on his legal authority as an agent of the Board of Health; he *orders* the child sent home, and at once reports the case to the Board of Health. He follows the case home and sees that it is properly isolated there, or is removed to the hospital. Later, when recovery is reported by the attending physician, he makes another visit to determine whether all danger of infection has ceased, and whether the patient shall be released from isolation. In all this he is acting under the law and for the public safety.

The great advantage arising from his visits to the schools is the early discovery of symptoms of infectious disease among the children. Such early discovery and the prompt measures thereupon taken have resulted in putting a stop to epidemics that could easily have become, through neglect, widespread and disastrous.

Accounts of epidemics arrested in this way have been published in medical journals. From one such account, written by a medical inspector, I copy a few paragraphs to show how an epidemic of diphtheria was handled. It started in a first (lowest) grade primary class, and had made considerable progress before any cases had become known to the teacher or had been reported to the Board of Health. The inspector, noticing that the first two cases reported to him came from the same school, proceeded at once to investigate. He found the teacher already alarmed by finding on the Board of Health report for that morning two more cases of diphtheria from her class, by rumors, of two or three other cases among the absent children, and by finding three cases of sore throat in the room. The inspector goes on to say:

> It was at once apparent to me that we had an epidemic on hand. Four suspicious cases were sent home and recommended to the care of the family physician The extent of the epidemic was investigated during the day ; the Board of Health had arrangements made for disinfection at nine the next morning ; the class was allowed to assemble for purpose of investigation, every throat being examined, and a culture taken in all suspicious cases, the class was then dismissed, and the formaldehyde generators set to work at once. This was on Thursday morning. After disinfection the room was cleaned up, allowed to air for two days, and opened for school again on Monday. For a week and a half the throats of all pupils present in the class were examined the first thing in the morning. No child who had any suspicious symptoms was allowed to return until a culture from the throat was found to be negative. As a result of these measures, notwithstanding the fact that the epidemic had made such good headway before discovery, not a single case of diphtheria developed after the epidemic was discovered except those known to have been infected at that time.

. . . . . . . . . . .

How little protection would come by trusting to the parents of some public school children is shown by their action in the following cases: four cases were sent home at the time of the discovery of the epidemic on the morning of May 5. They all had suspicious looking throats, and in view of the existing epidemic the diagnosis of diphtheria was warranted clinically. With each child was sent a note stating that there had been some cases of diphtheria in the school-room, that the child had a sore throat that probably was diphtheria, and that the school inspector advised calling the family physician. The mother of one of the children brought him back herself in the afternoon and was abusive to the teacher for sending him home. I turned up just in season to get my share. I was informed that I did not know what I was talking about, that his nausea and vomiting and constitutional symptoms two days before were due to indiscretion in eating, that his sore throat was simply "tonsillitis," that he was subject to, and that the patches in the throat were of no importance. The other children might have diphtheria, but *her* boy didn't, "because he wasn't sick enough." She refused to let me take a culture, and was informed that the boy would not be admitted to the school until a negative culture had been obtained by myself, or if she preferred by her family physician. She did not go to the family physician until another week had elapsed and a culture proved negative. I feel reasonably sure, however, that an earlier culture would have been positive.

The three other cases returned next morning. Two brought verbal messages that they were well enough to come to school. The third brought a note from his father saying it was absurd to say his boy had diphtheria. Cultures were taken from all three throats, and all proved positive. The mother of one of these was not convinced even then. She called another doctor to prove that the school doctor was wrong, and discharged him when he obtained a positive culture, and she was only partially persuaded there was no trick to it when a third doctor also found a positive reply to his culture.

One other case is instructive. This boy was subject to tonsillitis, so his parents did not attach any importance to a sore throat which began May 2. He returned May 5 and was examined. His throat was negative. I learned, however, that his brother who slept with him had a sore throat, beginning the day before, May 4, and had stayed at home, but that he also was at school that morning in another room. He was sent for, and I found several characteristic patches in his throat. Thus a possible source of infection of another class was nipped in the bud. Both were sent home with a note recommending the family physician, and stating what the probable trouble was. They did not consult a physician for ten days. Naturally the throats were cleared up then, and the physician, not understanding the full circumstances, gave them a note, stating that they might return to school. Admission was refused until a negative culture was found. They proved positive, and the one from the class-room which had the epidemic lasted positive for two weeks more. Had I allowed him to return without a culture he would have had two weeks in which to start another epidemic of diphtheria, which, while it might have been ascribed to imperfect disinfection of the room, would in reality have been due to my carelessness in taking it for granted that the throat was negative."—H. D. ARNOLD, M. D., *in Annals of Gynecology and Pediatry. Vol. XI. Boston, January, 1898.*

The same writer describes the suppression of an epidemic of scarlet fever which started with a pupil supposed by his parents to be ill with German measles, but found on investigation by the inspector to have scarlet fever.

In respect to infectious diseases, certainly the parents now have reason to feel that their children in school are much better protected than they would be without daily medical inspection. And as regards other diseases the earlier medical attention often secured in consequence of the inspector's visits is recognised as a great benefit. The masters of schools and the teachers have learned to depend upon the advice of the medical inspectors in various matters relative to the health of pupils and the sanitary condition of the schoolhouses.

The return of pupils after an absence for sickness, the attendance of pupils living in the same house, but not of the same household, with a person sick with an infectious disease, the admission of pupils without certificates of vaccination, the fitness as to the physical condition of certain children to be pupils of a school, and defects in ventilation and sanitation of the schoolhouses, are among the matters which receive attention from the medical inspectors, and concerning which they give advice. Generally speaking, such advice is gladly accepted and acted upon.

The following is an extract from the Manual of the Board of Education in reference to contagious diseases in public schools in New York:

1. Whenever the principal or teacher in charge of a school ascertains that a case of contagious disease has occurred in a family in which a child or children attending school lives or live, such child or children shall be immediately excluded from school; and a principal or teacher shall also exclude from school any child or children when notified by the Board of Health that the protection of public health requires it; and said child or children shall not be permitted to attend school until a certificate of the Board of Health is presented, stating that there is at the time no contagious disease existing in the family, and that it is safe for the child or children to attend school.

2. It shall be the duty of the teachers in the high and public schools, at the time of morning roll call, to select from their classes any child who appears to be ailing, or any child who, from any information they have received, they have reason to believe has been in contact, in its family or otherwise, with anyone ill with contagious or infectious diseases. Such children must be separated from the rest of the class, in a room set apart for that purpose by the principal, for examination by the medical school inspector at the time of his visit. The principal and teachers in charge of schools shall aid the inspector in such action as may be deemed necessary for the protection of the health of the other children of the school.

3. Whenever a pupil shall be dismissed from school on account of a sore throat or any eruptive disease, the principal or teacher in charge shall immediately notify the division of contagious diseases of such dismissal, and shall give in the report the name and address of the pupil and the disease causing the dismissal.

4. Whenever reports of the existence of contagious disease shall come to the principal or teacher in charge from other sources than the Board of Health, said report shall be communicated immediately to the division of contagious diseases.

5. New scholars applying for admission to school and living in houses in which a case of contagious disease has recently occurred or is then present, shall be admitted or excluded in compliance with the above rules.

6. The principal or teacher in charge shall make a weekly report to the Board of Health, on blanks provided for the purpose, of the names and addresses of all pupils absent from school on account of sickness of whatever nature, with the disease, so far as known, of those absent.

7. The floors, doors and door knobs, and the stairs and hand banisters of school-rooms shall be washed with a washing soda solution (one-half pound washing soda to three gallons of water) at least once each week.

8. When a pupil is excluded from school on account of any contagious disease, his or her desk and seat shall be washed with the soda solution.

9. Physicians shall be requested to notify the principal of the school which is attended by any person or any member of the family of a person suffering with contagious disease.

10. The division of contagious diseases shall send daily to the Board of Education a list of houses in which cases of contagious diseases have been reported during the previous twenty-four (24) hours, with the names, ages and diseases of the persons sick, and the Board of Education shall transmit such list the same day to every school under their charge.

11. A circular of information for pupils shall be prepared by the Board of Health for the Board of Education, to be printed, framed and hung in every school-room.

12. A circular of instruction for principals and teachers shall be prepared by the Board of Health and printed and distributed by the Board of Education.

By order of the Board of Health.

MICHAEL C. MURPHY,

*President.*

C. GOLDERMAN,

*Secretary pro tem.*

In the Chicago instructions "The pupils to be inspected will be referred to the inspector for two reasons: (1) Those who have been absent four or more consecutive days; (2) those in the school whom the principal may suspect to be suffering from contagious diseases."

In Philadelphia in 1900 the number of pupils enrolled in the public schools was 151,455. From January 1 to October 31, 1900, 3,446 cases of contagious disease were detected and 2,430 cases of non-contagious diseases were detected, *i.e.*, a little over 2¼ per cent. were suffering from contagious disease and a little less than 2 per cent. from other diseases, some of which were of sufficient seriousness to imperil the child's health—about 4 per cent. of those attending school were not in fit condition to do so. "The inspectors add that in their opinion epidemics have been averted by the early detection and exclusion of cases of disease and that a large number of pupils have been saved from illness by preventing the attendance at school of the sick, the uncleanly, and the vermin ridden."

In Chicago from January 8 to April 12, 1900, a little over 2½ per cent. of the school population was found suffering from contagious disease and was excluded from school.

Dr. Byrne says:

One of the most disagreeable duties of inspectors is to examine and exclude children afflicted with pediculi. One irate parent whose four children had been excluded from school kept after the writer for a month. During that time he complained to several members of the Board of Education, including its president. He also took the matter to the local alderman alleging every reason but the right one as the cause of exclusion. These men took the matter up but, on learning the truth, referred him back to his school principal and medical inspector. Finally he gave in and saw that his children had proper attention. They became models of cleanliness and were never again sent in for examination on that score."

According to the figures of the Chicago Health Department, as quoted by Dr. Byrne, covering diphtheria and scarlet fever for the year preceding medical inspection of schools and the first year under such inspection, the decrease in the number of cases of diphtheria was 628 and of scarlet fever 2,325, and that, too, in spite of the rapid growth of Chicago.

It is unnecessary to cite the figures from other cities: they all tell the same story and justify the conclusion of the Philadelphia inspectors that by the careful inspection of suspected and the exclusion of the sick from school, epidemics have been prevented and large numbers of pupils have been saved from illness.

This year in Buffalo there has been raging in the schools an epidemic of measles, and what steps have been taken to stop it? The Board of Health has no power in the matter except to exclude from attendance at school those cases reported to it; and all the cases are not reported. The teacher may send a pupil home if he thinks the child sick, but if the parent disagrees with the teacher, back the child comes at the next session. I have heard (uncorroborated rumor) of the closing of one department of one of the private schools for a short time on account of the epidemic, but so far as I know no department of any of the public schools has been closed. Necessarily, then, no disinfection of the rooms—much less of the buildings—has been attempted.

The accompanying table gives some interesting facts upon which it is worth while to ponder.

| | No. of Cases of Measles. | | Death from Measles. | |
|---|---|---|---|---|
| | 1900-1. | 1901-2. | 1900-1. | 1901-2. |
| September | 2 | 8 | 0 | 0 |
| October | 6 | 24 | 0 | 0 |
| November | 8 | 105 | 1 | 2 |
| December | 16 | 242 | 0 | 8 |
| January | 21 | 415 | 0 | 11 |
| February | 30 | 414 | 0 | 5 |
| March | 61 | 564 | 1 | 15 |
| April | 65 | 547 | 0 | 11 |
| Totals | 209 | 2,319 | 2 | 52 |
| May | 90 | 208 (½ month) | 3 | .. |
| June | 161 | .. | 6 | .. |
| July | 67 | .. | 1 | .. |
| August | 8 | .. | 1 | .. |

If we take the number of cases of measles reported each month from September, 1900, through April, 1902, we note that from September, 1900, to January, 1901, the number per month had increased from 2 to 21; that in February it reached 30; in March, 61; and by June had reached 161. Evidently the epidemic had started and we have a most convincing array of figures to show how thoroughly the schoolhouses had become infected, for with the closing of the schools the number of cases dropped to 67 in July and 8 in August. The schools begin in September. The number of cases reported in September was 8; in October, 24; in November, 105, and so on in steadily increasing number, reaching 564 in March, 547 in April and 208 for the first two weeks of May. In all there have been reported from September 1, 1901, to May 1, 1902, 2,319 cases of measles and 52 deaths from measles. In the same period in the previous year there were 209 cases of measles and 2 deaths from measles. The seriousness of the epidemic, however, is not represented by these figures. Measles is seldom a fatal disease, but it is apt to leave behind it catarrhal conditions of the eye and ear and of the respiratory and gastro-intestinal tracts that are sometimes serious in and of themselves and are especially serious as establishing a most excellent culture medium for the tubercle bacillus.

If the schools are only cleaned and not disinfected this summer, there remains the probability of more measles next year unless all the susceptible ones have already contracted the disease. If, however, so great an epidemic of measles can occur there is nothing to prevent one of scarlet fever with much more serious results or of diphtheria, similar to the one in Boston in 1894, which clinched the matter and induced the school board "to sanction medical inspection as a regular department of school life."

Is it necessary to wait for such a calamity, to sacrifice the health and jeopardise the lives of thousands of children before the proper steps are taken to prevent the occurrence of such epidemics? To recognise that a disease is infectious is to recognise that the spread of it is preventable. If preventable, there is no excuse for epidemics in modern civilised communities.

At the beginning of the establishment of medical inspection of schools there is always opposition on the part of some as there is to every reform movement, but when the people recognise the value of the inspection, often those who were most opposed are the staunchest supporters of the system.

In Chicago out of 76,805 examinations only one lawsuit instituted against the Board of Education by parents resulted, and in that instance Judge Ball of the Superior Court decided that the medical inspection of schools was constitutional and the rights of principals and medical inspectors to exclude pupils for cause were upheld. The case was never appealed.

Medical inspection has proved a great safeguard for the health of the children in Chicago public schools. The service has been handicapped by a limited number of inspectors and an inadequate appropriation to increase the force. By increasing the force the service could be improved, as inspectors now have too many schools to look after in their sub-districts.

The cost of the inspection is relatively slight considering the value of the result. In Chicago the figures of the Health Department, besides showing the reduction in number of cases already quoted show further that there were 46 fewer deaths from diphtheria and 307 fewer deaths from scarlet fever during the first year of inspection than in the preceding year when there was no inspection, 353 fewer deaths from these two diseases. If the low estimate of $1,000 is placed on a life we may say that, largely through medical inspection of schools, there was saved to the City of Chicago from the prevention of those two diseases only, the sum of $353,000.

The pay of the medical school inspectors in Chicago is $50 a month and their number is fifty, making a total expenditure of $2,500 per month or $22,500 for nine months. Remember that the figures quoted are for only two diseases. When we think of the other diseases and the other deaths prevented the cost of maintaining inspection is as nothing to the amount saved to the city through the maintenance of the system. As Dr. Byrne says: "To cover the ground properly a man must devote his entire working morning, from 9 a.m. to 12 m. to the service, each inspector visiting from 5 to 8 schools."

If the work is not done thoroughly it might as well be not done at all. Under the circumstances $50 a month seems a minimum fee and is certainly well earned. In Boston, however, there are fifty inspectors, appointed by the Board of Health, each of whom receives $200 per annum—about half the Chicago salary. There is a greater value in human life, however, than can be estimated in dollars and cents. No one knows that better than the physician who also knows well that in all human beings there is some good and in most human beings the goodness overbalances the evil mightily.

"Sana mens in corpore sano" should be the motto of all conscientious educators, and in their care of the children placed under their charge they should recognise the fact that if the moral goodness of the individual is to overcome any tendency to evil, the victory is much more easily won if there is a sound and healthy body in which and with which the soul may work. Therefore, the development and maintenance of the physical well-being of the individual is as much of our duty toward God as of our duty toward man. To love our fellowmen and to do unto others as we would they should do unto us is still the foundation of all true civilisation. It is our bounden duty, then, to provide for the maintenance of the health of the children, all possible safeguards by establishing the means of prevention of disease through the regular, daily medical inspection of schools.

Dr. F. Park Lewis, of this city, has already brought the matter to the notice of the clinical society and his paper was published in the *Sunday Express* a short time ago. For much that is of value in this paper I am indebted to him, as he put in my hands all the material he had collected, as well as his own valuable contribution. Can we not as physicians bring to the notice of the public the importance of this subject and thus through the pressure of public opinion bring about the establishment of medical inspection of schools in our city?

It has been said that "All things come to him who waits." Recognising some truth in that statement, let us pursue our enquiry a little further and learn *why* all things come to him who waits. It is because there are others who with restless energy are pursuing their ideals in the hope and firm belief of realising them. The waiter, with the others, enjoys the benefits obtained through the efforts of the worker.

> "Let *us* then be up and *doing*,
> With a heart for any fate,
> Still achieving, still pursuing,
> Learn to *labor* and to wait."

469 FRANKLIN STREET.

